# Sociodemographic determinants of multimorbidity in Brazilian adults and older adults: a cross-sectional study

**DOI:** 10.1590/1516-3180.2021.0105.R1.31052021

**Published:** 2022-01-17

**Authors:** Marina Christofoletti, Giovani Firpo Del Duca, Tânia Rosane Bertoldo Benedetti, Deborah Carvalho Malta

**Affiliations:** I MSc. Doctoral Student, Department of Physical Education, Universidade Federal de Santa Catarina (UFSC), Florianópolis (SC), Brazil.; II PhD. Full Professor, Department of Physical Education, Universidade Federal de Santa Catarina (UFSC), Florianópolis (SC), Brazil.; III PhD. Full Professor, Department of Physical Education, Universidade Federal de Santa Catarina (UFSC), Florianópolis (SC), Brazil.; IV PhD. Full Professor, Department of Maternal and Child Nursing and Public Health, Universidade Federal de Minas Gerais, Minas Gerais (MG), Brazil.

**Keywords:** Chronic disease, Diagnosis-related groups, Socioeconomic factors, Cross-sectional studies, Risk factors, Health surveys, Chronic illness, Social determinants, Epidemiology studies

## Abstract

**BACKGROUND:**

Multimorbidity due to non-communicable chronic diseases (NCDs) constitutes a significant challenge for healthcare systems. To attenuate its impacts, it is essential to identify the sociodemographic determinants of this condition, which can discriminate against population segments that are more exposed.

**OBJECTIVE:**

To identify associations between multimorbidity conditions and sociodemographic indicators among Brazilian adults and older adults.

**DESIGN AND SETTING:**

Cross-sectional telephone-based survey in 26 Brazilian state capitals and the federal district.

**METHODS:**

The Vigitel 2013 survey was used, with data collected via a questionnaire. The outcome was multimorbidity (2, 3 or 4 NCDs), and the exposures were sociodemographic indicators (age, sex, skin color, marital status and education). The analysis consisted of multinomial logistic regression (odds ratio), stratified by age.

**RESULTS:**

Among adults, multimorbidity comprising two, three or four diseases was associated with advancing age (P < 0.001); two and three diseases, with having a partner (P = 0.004 and P < 0.001, respectively); and two, three or four diseases, with lower education (P < 0.001). Among older adults, two, three or four diseases were associated with female sex (P < 0.001); three diseases, with living with a partner (P = 0.018); two diseases, with black skin color (P = 0.016); and two or three diseases, with lower education (P < 0.001).

**CONCLUSIONS:**

To control and prevent multimorbidity, strategies for individuals with existing chronic diseases, with partners and with lower education levels are needed. Particularly for adults, advancing age should be considered; and for older adults, being a woman and having black skin color.

## INTRODUCTION

Multimorbidity due to non-communicable chronic diseases (NCDs) constitutes a significant challenges for healthcare systems.^1^It is reflected in increased health problems, financial expenditure and decreased quality of life.^2^Over 95% of the population over 65 years of age is estimated to present more than one disease diagnosed in primary healthcare.^3^Middle-income countries such as China,^4^Serbia, Mexico, Russia, South Africa and India^5^present alarming multimorbidity results, but knowledge about simultaneous diseases in Latin American countries is limited. Specifically in Brazil,^6^studies have emphasized occurrences in subgroups of the population comprising older adults.^7–9^

Investigation of the determinants of multimorbidity, especially the sociodemographic aspects, is crucial for planning preventive public policies.^10^Some important variables need to be included in these investigations, in order to identify the population segment that is more exposed to NCD multimorbidity. These variables may include sex, age, marital status, skin color and educational level. The locality is also important. It can also be considered that population density, seasonal patterns, urbanization, economy^11^and cultural contextualization may influence inequalities and health disparities.

Separately, each NCD has important sociodemographic determinants, which reflect health inequalities and the populations most affected. In the case of multimorbidity, identifying the inherent indicators of the population is of considerable importance, in order to reflect on phenomena of disability and mortality, which are strongly related to diagnoses of multiple chronic diseases.^2^Therefore, recognition of sociodemographic determinants enables knowledge of the population subgroups that are most vulnerable to the aggravation of presenting interactions of multiple diseases.

## OBJECTIVE

The aim of the present study was to identify the sociodemographic factors associated with multimorbidity due to non-communicable chronic diseases among adults and older adults in Brazil.

## METHODS

### Design and sample

The data for this cross-sectional study came from the annual national survey “Surveillance of Risk Factors and Protection Against Chronic Diseases by Telephone Inquiry” (Vigitel in the Portuguese-language acronym), conducted between February and December 2013. The sampling process was performed in three steps.^12^Weighting factors were used to compensate for nonuniversal fixed-line coverage bias, adjusted for the adult Brazilian population. This was based on each individual's weight in the sample, calculated via the ranking method. Details about the methodology, including the sampling process, weighting factors and ethics procedures are provided in the official report.^12^

### Sample

The participants were a representative sample of adults (≥ 18 years old) living in all 27 Brazilian state capitals. To be eligible, the requirement was that the participant needed to have a residential landline telephone.

### Data collection

Data were collected by means of telephone interviews simultaneously using a computer, and all the calls were recorded in case any queries arose. The instrument used was a validated questionnaire, and it was applied via telephone calls by trained staff. The questionnaire asked about sociodemographic, behavioral, nutritional and health factors.

### Measurements

The outcome variable was multimorbidity due to non-communicable diseases (NCDs). The concept investigated was co-occurrence of multiple chronic or acute diseases and medical conditions within a single person, without an index condition, giving equal attention to all diagnoses.^13^The four categories proposed were: no occurrence (zero or one disease), and multimorbidity as occurrences of two diseases, three diseases or four diseases. The diseases considered were the four most prevalent NCDs in Brazil (diabetes, dyslipidemia, arterial hypertension and obesity). Occurrences were considered to consist of affirmative self-reports of diabetes, dyslipidemia or arterial hypertension; and for obesity, body mass index (BMI) of 30 kg/m² or higher (calculated based on self-reported weight and height). For BMI, hot-deck imputation of the data was used. The exposure variables used were sex (male or female), age groups (adult categories: 18 to 29, 30 to 39, 40 to 49 or 50 to 59 years old; older adult categories: 60 to 69, 70 to 79 or 80 years old and over), marital status (living without a partner or living with a partner), ethnicity (white or black) and education level (less than eight, nine to eleven or twelve years and over) and demographic macroregion (center-west, northeast, north, southeast and south).

### Analyses

The analysis, conducted in 2016, was stratified according to age group (adults: 18 to 59 years; and older adults: ≥ 60 years). The descriptive analyses comprised absolute and relative frequencies, considering prevalence estimates and 95% confidence intervals (95% CI). Multinomial logistic regression (odds ratio [OR]) formed the inferential analysis that was used to investigate associations of sociodemographic indicators with each multimorbidity category (taking zero or one disease as the reference category). The first-level variables were sex, age, marital status, ethnicity and demographic macroregion; and the second level was education level. Backward selection was adopted for statistical modeling, with a critical level of P ≤ 0.20 for each variable to remain in the hierarchical regression model, so as to minimize the confounding control. The significance level was 5% for all tests. All analyses considered sample weighting obtained through the inverse of the number of telephone lines existing in the household that was interviewed and the number of adults living in the interviewee's home.

### Ethics

The Brazilian Ministry of Health's National Ethics Committee for Research on Human Beings approved this study under registration number 355.590/2013, on June 26, 2013.

## RESULTS

Within the response rate of 71.5%, the total number of participants was 52,929 (37,947 adults and 14,982 older adults). Among the adults, the following were predominant: females (52.9%), individuals living without a partner (52.0%), blacks (55.1%), individuals with nine to eleven years of schooling (41.1%) and those living in the southeastern region (44.1%). Among the older adults, the following were predominant: females (59.5%), individuals living with a partner (56.9%), those of white non-Hispanic ethnicity (61.3%), those eight or fewer years of schooling (69.3%) and those living in the southeastern region (51.8%) (Table 1).

**Table 1:** Sociodemographic and health characteristics stratified according to age. Brazil, 2013 (n = 52,929)

Variables		Adults (n = 37,947)				Older adults (n = 14,982)			
		n	%^a^	95% CI^b^	% missing	n	%^a^	95% CI^b^	% missing
**Sex**					0.0				0.0
	Male	15,368	47.1	(46.2; 48.1)		4.908	40.5	(38.7; 42.2)	
	Female	22,579	52.9	(51.9; 53.8)		10.074	59.5	(57.8; 61.3)	
**Marital status**					1.1				1.5
	Living without a partner	18,210	52.0	(51.0; 53.0)		7.557	43.1	(41.4; 44.8)	
	Living with a partner	19,304	48.0	(47.0; 49.0)		7.204	56.9	(55.2; 58.6)	
**Skin color**					1.1				18.6
	White	14,867	44.9	(43.8; 45.9)		7.402	61.3	(59.4; 63.2)	
	Black	18,983	55.1	(54.1; 56.2)		4.790	38.7	(36.8; 40.6)	
**Educational level, years**					0.8				2.9
	0-8	7,115	30.6	(29.6; 31.6)		7.405	69.3	(67.8; 70.7)	
	9-11	15,532	41.1	(40.2; 42.0)		3.761	17.3	(16.3; 18.5)	
	≥ 12	14,984	28.3	(27.5; 29.2)		3.381	13.4	(12.4; 14.4)	
**Demographic macroregion**					0.0				0.0
	North	10,896	10.5	(10.2; 10.9)		2.805	7.0	(6.6; 7.5)	
	Northeast	12,729	25.7	(25.0; 26.3)		4.884	22.2	(21.1; 23.2)	
	Southeast	5,327	44.1	(43.1; 45.2)		2.574	51.8	(50.1; 53.5)	
	South	3,457	7.9	(7.5; 8.2)		2.399	9.5	(8.9; 10.1)	
	Center-West	5,538	11.8	(11.3; 12.2)		2.320	9.5	(8.9; 10.1)	

95% CI = 95% confidence interval.

a Values weighted for the inverse of existence of landline telephones and the number of adults living in the household of the interviewee

b 95% confidence interval in weighted sample.

**Figure 1:**
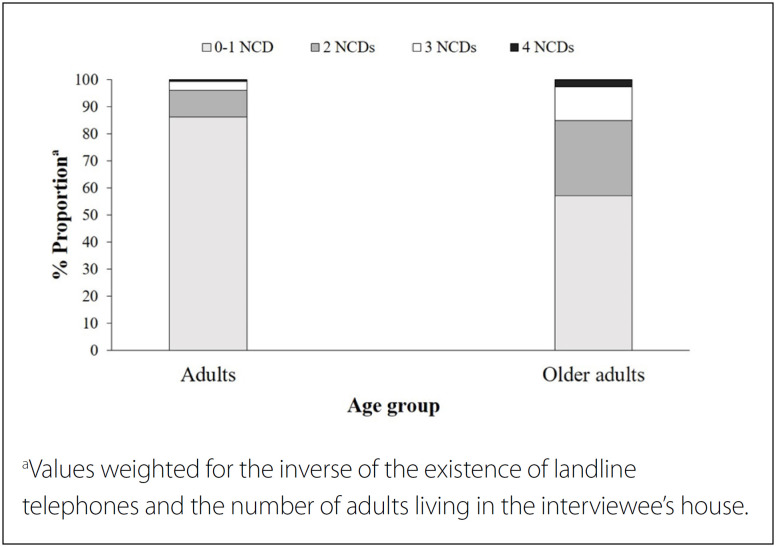
The proportionᵃ of concomitant non-communicable chronic diseases (NCDs), according to age groups. Brazil, 2013 (n = 52,929).

The frequency of multimorbidity in adults was 13.7% (9.8% with two, 3.3% with three and 0.6% with four NCDs), and 42.9% among old adults (27.9% with two, 12.4% with three and 2.7% with four NCDs) (Figure 1).

Tables 2and 3indicate the associations between sociodemographic variables and multimorbidity categories. In Table 2, for multimorbidity consisting of two chronic diseases, the odds increased according to the advancement of the age group (40 to 49 years, OR: 7.07 [CI: 5.67; 8.82]; 50 to 59 years, OR: 13.71 [CI: 11.04; 17.03]) and were also higher for individuals living with a partner (OR: 1.27 [CI: 1.08; 1.50]). On the other hand, the odds decreased with higher educational levels (9 to 11 years, OR: 0.73 [0.62; 0.86]; 12 years and over, OR: 0.67 [CI: 0.56; 0.81]), in comparison with adults with no and one disease. For multimorbidity consisting of three diseases, the odds increased according to the advancement of the age group (40 to 49 years, OR: 11.44 [CI: 6.27; 20.86]; 50 to 59 years, OR: 28.57 [CI: 15.90; 51.34]) and were also higher for individuals living with a partner (OR: 1.70 [CI: 1.28; 2.26]). The odds decreased with higher educational levels (9 to 11 years, OR: 0.57 [CI: 0.43; 0.74]; 12 years and over, OR: 0.47 [CI: 0.34; 0.64]), in comparison with adults with no and one disease. The odds for this group were also associated with the demographic macroregion, indicating that the southeastern and southern regions were more exposed than the other demographic macroregions. For the last category, multimorbidity consisting of four diseases, the odds increased only according to the advancement of the age group (40 to 49 years, OR: 13.76 [CI: 3.49; 54.21]; 50 to 59 years, OR: 30.01 [CI: 8.01; 112.5]) and decreased with higher educational level (9 to 11 years, OR: 0.40 [CI: 0.22; 0.74]; 12 years and over, OR: 0.30 [CI: 0.15; 0.60]), in comparison with adults with no and one disease. It can be seen that the odds ratio increased with increasing numbers of NCDs, thus expressing a potential profile.

**Table 2:** Association*of sociodemographic indicators with multimorbidity in terms of the number of non-communicable chronic diseases (NCDs), among adults (n = 37,947)

Variables		2 NCDs versus 0 and 1		3 NCDs versus 0 and 1		4 NCDs versus 0 and 1	
		OR (95% CI)	P	OR (95% CI)	P	OR (95% CI)	P
**Sex**			0.641^a^		0.775^a^		0.841^a^
	Male	1.00		1.00		1.00	
	Female	0.94 (0.83; 1.12)		0.96 (0.74; 1.25)		1.06 (0.60; 1.88)	
**Age group, years**			< 0.001^b^		< 0.001^b^		< 0.001^b^
	18-29	1.00		1.00		1.00	
	30-39	4.36 (0.44; 5.52)		5.00 (2.62; 9.44)		1.50 (0.34; 6.59)	
	40-49	**7.07 (5.67; 8.82)**		**11.44 (6.27; 20.86)**		**13.76 (3.49; 54.21)**	
	50-59	**13.71 (11.04; 17.03)**		**28.57 (15.90; 51.34)**		**30.01 (8.01; 112.5)**	
**Marital status**			0.004^a^		< 0.001^a^		0.889^a^
	Living without a partner	1.00		1.00		1.00	
	Living with a partner	**1.27 (1.08; 1.50)**		**1.70 (1.28; 2.26)**		1.04 (0.77; 2.64)	
**Skin color**			0.230^a^		0.226^a^		0.264^a^
	White	1.00		1.00		1.00	
	Black	1.10 (0.94; 1.29)		1.18 (0.90; 1.55)		1.42 (0.77; 2.65)	
**Educational level, years**			< 0.001^b^		< 0.001^b^		< 0.001^b^
	0-8	1.00		1.00		1.00	
	9-11	**0.73 (0.62; 0.86)**		**0.57 (0.43; 0.74)**		**0.40 (0.22; 0.74)**	
	≥ 12	**0.67 (0.56; 0.81)**		**0.47 (0.34; 0.64)**		**0.30 (0.15; 0.60)**	
**Demographic macroregion**			0.638^a^		0.097^a^		0.638^a^
	North	1.00		1.00		1.00	
	Northeast	1.07 (0.91; 1.26)		1.22 (0.88; 1.68)		0.97 (0.51; 1.83)	
	Southeast	1.02 (0.85; 1.22)		**1.45 (1.00; 2.09)**		1.49 (0.78; 2.87)	
	South	1.03 (0.84; 1.25)		**1.52 (1.06; 2.17)**		1.13 (0.54; 2.34)	
	Center-West	0.92 (0.74; 1.15)		1.02 (0.68; 1.55)		1.17 (0.53; 2.59)	

OR = odds ratio; 95% CI = 95% confidence interval; P = significance level.

a Heterogeneity

b Tendency.

* Values weighted for the inverse of the existence of landline telephones and the number of adults living in the interviewee's house.

Analysis adjusted for sex, age, marital status, skin color and demographic macroregion (first level), and education level (second level).

Boldface indicates statistical significance (P < 0.05).

**Table 3:** Association*of sociodemographic indicators with multimorbidity in terms of the number of non-communicable chronic diseases (NCDs), among older adults (n = 14,982)

Variables		2 NCDs versus 0 and 1		3 NCDs versus 0 and 1		4 NCDs versus 0 and 1	
		OR (95% CI)	P	OR (95% CI)	P	OR (95% CI)	P
**Sex**			**< 0.001** a		**< 0.001** a		**0.002** a
	Male	1.00		1.00		1.00	
	Female	**1.62 (1.33; 1.96)**		**1.68 (1.28; 2.21)**		**2.52 (1.39; 4.57)**	
**Age group, years**			0.420^b^		0.356^b^		0.143^b^
	60 to 69	1.00		1.00		1.00	
	70 to 79	1.10 (0.89; 1.37)		1.33 (0.98; 1.81)		0.65 (0.39; 1.10)	
	≥ 80	0.76 (0.56; 1.04)		1.03 (0.61; 1.75)		0.57 (0.21; 1.58)	
**Marital status**			0.580^a^		**0.018** a		0.278^a^
	Living without a partner	1.00		1.00		1.00	
	Living with a partner	1.06 (0.85; 1.33)		**1.45 (1.06; 1.98)**		0.74 (0.43; 1.28)	
**Skin color**			**0.016** a		0.380^a^		0.814^a^
	White	1.00		1.00		1.00	
	Black	**1.31 (1.05; 1.62)**		1.14 (0.86; 1.51)		0.94 (0.59;1.52)	
**Educational level, years**			**< 0.001** b		< **0.001**^b^		0.369^b^
	0-8	1.00		1.00		1.00	
	9-11	**0.68 (0.56; 0.83)**		**0.58 (0.45; 0.77)**		0.73 (0.38;1.38)	
	≥ 12	**0.63 (0.50; 0.79)**		**0.49 (0.35; 0.69)**		0.79 (0.40; 1.57)	
**Demographic macroregion**			0.581^a^		0.327^a^		0.504^a^
	North	1.00		1.00		1.00	
	Northeast	0.07 (0.87; 1.31)		1.04 (0.77; 1.40)		0.70 (0.42; 1.17)	
	Southeast	0.92 (0.72; 1.17)		1.00 (0.70; 1.43)		**0.52 (0.28; 0.97)**	
	South	1.18 (0.93; 1.49)		1.24 (0.89; 1.73)		0.93 (0.54; 1.61)	
	Center-West	0.92 (0.74; 1.15)		0.97 (0.71; 1.33)		**0.37 (0.20; 0.69)**	

OR = odds ratio; 95% CI = 95% confidence interval; P = significance level.

a Heterogeneity

b Tendency.

* Values weighted for the inverse of the existence of landline telephones and the number of adults living in the interviewee's house.

Analysis adjusted for sex, age, marital status, skin color and demographic macroregion (first level), and education level (second level).

Boldface indicates statistical significance (P < 0.05).

Among older adults, being female was associated with two, three and four diseases (OR: 1.62 [CI: 1.33; 1.96]; OR: 1.68 [CI: 1.28; 2.21]; and OR: 2.52 [CI: 1.39; 4.57], respectively). For multimorbidity consisting of two diseases, the odds increased among individuals with black skin color (OR: 1.31 [CI: 1.05; 1.62]) and decreased according to the educational level (9 to 11 years, OR: 0.68 [CI: 0.56; 0.83]; 12 years and over, OR: 0.63 [CI: 0.50; 0.79]), in comparison with adults with no and one disease. For multimorbidity consisting of three diseases, the odds increased among individuals living with a partner (OR: 1.45 [CI: 1.06; 1.98]) and decreased according to the educational level (9 to 11 years, OR: 0.58 [CI: 0.45; 0.77]; 12 years and over, OR: 0.49 [CI: 0.35; 0.69]), in comparison with adults with no and one disease. Lastly, an inverse association between multimorbidity consisting of four diseases and living in the southeastern and central-western demographic macroregions was also observed (Table 3).

## DISCUSSION

In this study, the aim was to identify sociodemographic factors associated with multimorbidity due to non-communicable chronic diseases among adults and older adults in Brazil. Among adults, the main sociodemographic factors related to multimorbidity due to two, three and four chronic diseases were age between 40 to 59 years, the lowest educational level and the fact that they lived in the southern and southeastern regions. Among older adults, being female and having the lowest educational level were the characteristics that presented the most consistent associations with occurrences of multimorbidity, while residing in the southeastern and central-western regions was an inverse feature.

In the present study, older women were at greater risk of NCD multimorbidity. However, among adults, this same result was not seen. In a review of the literature by Marengoni et al.,^14^on the multimorbidity process in relation to aging, it was indicated that women characterized this state of health more clearly. Another study identified higher risk for women regardless of age, with a magnitude lower than in the present study (OR = 1.12).^15^The higher risk presented by women in this age group can be attributed to the postmenopausal period^16^and to the fact that women have greater knowledge of medical diagnoses.^17^

Advancement of age among adults presented a tendency to increase the risk of accumulation of NCD multimorbidity. On the other hand, among the older adults, there was no association with this variable. The results from the present study were in line with findings from the United States,^18^Australia^19^and middle-income countries such as China, Ghana, India, Mexico, Russia and South Africa.^20^Especially between the ages of 50 and 60 years, the aging process is associated with significant transitions that give rise to diminished autonomy, mental health, quality of life and physical functioning.^21^According to Willcox, Ash and Catignani,^22^aging can be attributed to genetically engineered mechanisms, neuronal-endocrine failures and modifications resulting from the oxidative stress proteins and deoxyribonucleic acid (DNA) of cellular lipids. These alterations increase chronic inflammatory states, since they are not controlled mainly by behavioral factors, and they therefore exposed personal effects to the appearance of NCDs.^22^

Considering marital status, the adults and the older adults with partners were at greater risk of having multimorbidity consisting of two and three NCDs, respectively. Other studies have also found this association of risk among adults,^4,23^which can be attributed to changes to unhealthy habits, with increasing burden of disease appearing over the course of the routine that characterizes marital transition.^24^Evidence for this association had already been presented,^25^but assessment of multimorbidity consisting of concomitant diseases constitutes a new approach. This may be explained differently among adults, in the light of the transition of the marital situation. Another interpretation that might be suggested is that individuals with a partner are more likely to use healthcare services than are those without a partner.^26^

The adults’ skin color did not show any association with multimorbidity, but among the older adults, there was higher risk of multimorbidity with two NCDs among individuals with black skin color. This result can be characterized in social or biological terms. Socially, older adults with black skin color have social, economic and cultural barriers regarding their living and health conditions, which reflect the unequal distribution of risk factors, protective factors and health problems accumulated over the course of their lives.^27^From a biological point of view, people with black skin color present higher risk of hypertension,^28^which leads to higher exposure to new diseases concomitantly, i.e. multimorbidity.

Adults with more years of schooling presented protection against multimorbidity, especially with regard to dealing with four diseases simultaneously. The same association was also found among older adults, but in relation to occurrences of two and three NCDs. Other studies investigating the association of schooling with multimorbidity have also found protection, especially in adulthood.^4^This result can be attributed to these individuals’ knowledge of disease prevention, health promotion measures and care and control measures after a disease has become established. Actions towards providing such care and management would, for example, change modifiable factors associated with NCDs and would entail correct use of medications. A trend towards protection was also found in populations older than 60 years of age in Scotland^1^and the United States,^23^among individuals with higher educational levels. Longer schooling reflects better social conditions, with consequent indication of characteristics favoring access to information and healthcare services. A study by Bosma et al.^29^made this relationship clear by pointing out that higher educational levels among older adults allowed health self-care interventions to be more effective.

Lastly, regarding the demographic macroregions of Brazil, there was a risk of multimorbidity with two NCDs among adults living in the southeastern and southern regions, while there was protection with four NCDs in the southeastern and central-western regions among older adults. Brazil is a country of continental dimensions with diverse settlements, demographics, climates and cultures, which lead to peculiar features with regard to data interpretation. For adults, the southeastern and southern regions may present greater risk because areas with more urban lifestyles present greater inequalities in healthcare and more discrepant socioeconomic indicators, which are considered to be a proxy for multimorbidity.^3^The counterpoint described for the older adult population can be explained by the higher proportion of financial resources that are allocated to primary healthcare in the southeastern and central-western regions of Brazil,^30^which enables better prevention and control measures regarding health outcomes, including NCDs.

We recognize that there were some limitations to this study that need to be considered before interpreting the results. The main limitation was the number of NCDs included as multimorbidities. This hampers comparisons with data from other countries or from using different instruments. Moreover, the measurements were self-reported, which required that the interviewees were aware that the disease had been diagnosed. The fact that Brazil's public healthcare programs give the population broad access to primary healthcare also needs to be considered.^31^Our study did not assess the contributions that may have been made by substantial numbers of other diseases and their severity.

Furthermore, the obesity classification was based on self-reported weight and height information and should be considered cautiously. Lastly, even though the sample was representative nationally, it is necessary to restrict the extrapolation of the results. Only individuals residing in the capitals of the Brazilian states and those who had landline telephones were considered, although the weighting used in Vigitel aimed to minimize this source of bias.

## CONCLUSION

The numbers of diseases in the Brazilian population among adults and older adults have sociodemographic determinants with regard to defining multimorbidity. The variables presented differences in the magnitudes of effect in analyses on the age groups of adults and older adults. The disparity among adults was in relation to the advancement through the decades of life; and among older adults, in relation to being female. These findings are significant because they relate to a current primary healthcare topic, aimed mainly at public healthcare in Brazil. The future implications may be contained in preventive and therapeutic multicomponent care programs within primary healthcare in Brazil and other middle-income countries. This possibility allows actions based on construction of informational materials, training of professionals and organization of activities directed to women's population, individuals of older age, those living with a partner and those with lower educational levels.
